# Nasal Discharge.—III

**Published:** 1909-06-19

**Authors:** 


### Diseases of Children.

### NASAL DISCHARGE.—III.

*Hypertrophic rhinitis* is a rare cause of bilateral discharge, but it may follow on recurrent coryza or adenoids. It affects all the mucosa, especially that of the inferior turbinals, and leads to chronic catarrh and bilateral stenosis. Some cases can be cured by tonics and local treatment. The galvano-cautery is useful, and pendulous masses can be removed by snares. *Atrophic rhinitis* is almost unknown under twelve years of age. It may be due to congenital syphilis, and has ensued on myiasis.

*A Unilateral Discharge* is generally due to a foreign body, occasionally to myiasis, nasal polypus, tumours, tuberculosis, rhinitis caseosa, some of the affections which give rise to bilateral discharge, and rarely in children to disease of the antrum, frontal, or ethmoid sinuses.

*Foreign Bodies* are the main cause, and the nature of the discharge depends more on the kind of foreign body than the duration of its presence. At first it merely causes a little mechanical irritation, perhaps none at all if it is a smooth bead or button. Thus, a button during a period of six months only caused a slight discharge; a disagreeable one for three years was due to a cherry stone, which became covered with a fibrinous deposit; while a stinking discharge for six weeks was due to a piece of window curtain (H. C. Cameron). Commonly the discharge is very chronic, profuse, muco-purulent or purulent, often blood-stained, and excoriates the nares. The body is nearly always in the inferior meatus. In recent cases by exciting sneezing or getting the child to blow strongly, with the unaffected nostril compressed, a smooth foreign body can often be evacuated. If not, apply cocaine as an astringent and anæsthetic, stand behind the child, and push a probe or director gently along the floor of the nose, thus displacing the body upwards. Then, using the margin of the floor as a fulcrum for the director, push the object upwards and forwards, and it will probably glide downwards and out. A general anæsthetic may be needed. Subsequently apply alkaline lotions, with a disinfectant if necessary. Syringing is unsuitable treatment, for it may drive septic matter up the Eustachian tube.

*Myiasis* (maggots) is due to the meat fly, *musca vomitaria*, laying its eggs in a fold of mucous membrane. An acute rhinitis is set up and may end in ulceration, perforation of the septum, or even fœtid atrophic rhinitis. The maggots can be killed by chloroform vapour, or by warm olive oil, liquid vaseline and such like oily preparations, which suffocate them by blocking up the pores or stigmata of the respiratory system. They can then be syringed out.

A *nasal polypus*, mucus or fibrous, is rare. It causes unilateral obstruction and serous discharge; sometimes bilateral symptoms, occasionally headache, sneezing, cough, and asthma. It is removed by snare or forceps. Of *tuberculous disease* there are only about eight cases on record. It may cause a large tumour of granulation tissue, resembling sarcoma, and ulceration and destruction of the cartilaginous septum. The diagnosis depends on microscopical examination. It is treated by curettage.

*Rhinitis caseosa* is also rare and almost invariably unilateral. It is probably due to chronic suppuration and retention of pus, with subsequent changes in the pus which becomes caseous, putty-like, and fœtid. Various organisms have been found. It is somewhat like the tuberculous affection, but there are no tubercle bacilli, and it differs from cholesteatoma in the absence of cholesterin.

*Mode of Examination.*—Complete examination is often difficult because of the small size of the nose and the nervousness of the child. Simply elevating the tip of the nose and lifting the ala upwards and outwards with a probe gives a good view of the interior meatus and may enable a diagnosis to be made of foreign body, diphtheria, sinus disease, etc. Often a mirror and a nasal speculum are needed.

*Diagnosis* depends on the results of examination; the history—*e.g.* insertion of a foreign body, congenital syphilis, exposure to diphtheria, influenza, measles, etc.; the nature of the discharge. Chronicity favours adenoids or syphilis. Very long duration indicates a foreign body.

Thick muco-pus generally means adenoids or syphilis. A blood-stained discharge is likely to be due to syphilis, diphtheria, or a foreign body. Blood and pus may be present in tuberculous disease. Pure liquid pus is due occasionally to a foreign body, but more commonly to sinus suppuration. The pus is liquid, yellowish, has a peculiar odour, is seen above the inferior turbinate bone and flows over the upper part towards the septum. In atrophic rhinitis the discharge is mucoid and watery. Large brownish or greenish-grey crusts and plugs are formed, and there is distinct ozæna. The nasal cavity is large and the mucous membrane atrophied. In rare cases a profuse watery discharge has been due to the escape of cerebro-spinal fluid by the nose. The general treatment of all nasal affections is by cleanliness, using alkaline lotions to dissolve the mucoid secretion and evacuate it, mild disinfectants if the discharge is at all offensive, and the removal of any local cause, such as adenoids and foreign bodies.

*Prophylaxis* is of little value, except in the prevention of simple recurrent acute coryza, and for chronic coryza in fat flabby children. Moderate starvation, a reduction in the amount of carbohydrate food, and an increase in fats and undercooked meat, is beneficial. The other measures include fresh air, outdoor life, sea air, brine baths, exercise, and suitable clothing. Judicious hardening is essential. Avoid coddling. Iron preparations, the iodide of iron, cod-liver oil, arsenic, iodine in some form, hypophosphites, and possibly phosphorus, are the drugs most valuable for improving the general health. It is of the utmost importance to guard infants during the first year of life, especially if at all delicate, from exposure to the infection of the influenza cold, true influenza, measles, and diphtheria. Moreover, the mildest attack of nasal catarrh should be treated, for its secondary effects, if progressive, are liable to be exceedingly dangerous.

